# Venomous Landmines: Clinical Implications of Extreme Coagulotoxic Diversification and Differential Neutralization by Antivenom of Venoms within the Viperid Snake Genus *Bitis*

**DOI:** 10.3390/toxins11070422

**Published:** 2019-07-19

**Authors:** Nicholas J. Youngman, Jordan Debono, James S. Dobson, Christina N. Zdenek, Richard J. Harris, Bianca op den Brouw, Francisco C. P. Coimbra, Arno Naude, Kristian Coster, Eric Sundman, Ralph Braun, Iwan Hendrikx, Bryan G. Fry

**Affiliations:** 1 Venom Evolution Lab, School of Biological Sciences, University of Queensland, St Lucia, QLD 4072, Australia; 2 Snakebite Assist, Pretoria ZA-0001, South Africa; 3 Universeum, Södra Vägen 50, 412 54 Gothenburg, Sweden; 4 Serpentarium Calden, Birkenweg 11, 34379 Calden, Germany

**Keywords:** *Bitis*, coagulotoxicity, anticoagulant, procoagulant, snakebite

## Abstract

The genus *Bitis* comprises 17 snake species that inhabit Africa and the Arabian Peninsula. They are responsible for a significant proportion of snakebites in the region. The venoms of the two independent lineages of giant *Bitis (B. arietans* and again in the common ancestor of the clade consisting of *B. gabonica, B. nasicornis, B. parviocula* and *B. rhinoceros*) induce an array of debilitating effects including anticoagulation, hemorrhagic shock and cytotoxicity, whilst the dwarf species *B. atropos* is known to have strong neurotoxic effects. However, the venom effects of the other species within the genus have not been explored in detail. A series of coagulation assays were implemented to assess the coagulotoxic venom effects of fourteen species within the genus. This study identified procoagulant venom as the ancestral condition, retained only by the basal dwarf species *B. worthingtoni,* suggesting anticoagulant venom is a derived trait within the *Bitis* genus and has been secondarily amplified on at least four occasions. A wide range of anticoagulant mechanisms were identified, such as coagulant and destructive activities upon fibrinogen in both giant and dwarf *Bitis* and the action of inhibiting the prothrombinase complex, which is present in a clade of dwarf *Bitis*. Antivenom studies revealed that while the procoagulant effects of *B. worthingtoni* were poorly neutralized, and thus a cause for concern, the differential mechanisms of anticoagulation in other species were all well neutralized. Thus, this study concludes there is a wide range of coagulotoxic mechanisms which have evolved within the *Bitis* genus and that clinical management strategies are limited for the procoagulant effects of *B. worthingtoni*, but that anticoagulant effects of other species are readily treated by the South African polyvalent antivenom. These results therefore have direct, real-work implications for the treatment of envenomed patients.

## 1. Introduction

Snakebite envenomations are one of the most neglected tropical diseases globally [1,2]. Envenomations are complex medical emergencies, as the venom affects any part of the physiological system reachable by the bloodstream. In Africa, species in the viperid genus *Bitis* are the most widespread venomous snakes across the continent and are responsible for a substantial percentage of the snakebite mortality and morbidity rates across Africa [1,2]. 

Seventeen species are recognized within the *Bitis* genus, which are found throughout a wide range of habitats such as deserts, rocky outcrops, rainforests, marshlands and coastal dunes [3,4,5]. Species within the genus are split into two different morphological groups: the dwarf *Bitis* (less than 50cm in length); and the giant *Bitis* (up to and exceeding 2m in length) [4,5]. Phylogenetic studies strongly suggest that dwarf species retain the basal condition, closely resembling the sister genus *Proatheris,* and that gigantism has evolved on two separate occasions within the genus *(B. arietans* and again in the common ancestor of the clade consisting of *B. gabonica, B. nasicornis, B. parviocula,* and *B. rhinoceros*) [6,7,8,9]. Despite their significant medical importance and unique evolutionary and morphological attributes, no detailed studies have investigated the differential venom effects across the *Bitis* genus and the clinical or ecological relevance of any variation or link to either of the morphological conditions (dwarf and giant) within the genus.

The venom composition of snakes is under extreme selection pressure and is dependent on a variety of factors, primarily prey type and prey escape potential [10,11,12]. Diet is thought to be a main driver in snake venom evolution, as immobilization of prey is the primary function of venom [10,11,13]. Venom composition seems to reflect the diet specialization of a species, with venoms having prey-specific toxic effects [13,14]. 

Although significant research has focused on the neurotoxic effects of venoms, comparatively little work has been done on the coagulotoxic effects of snake species due to the inherent difficulties in working with blood without the use of advanced robotics such as that utilized in this study [15,16,17]. Venoms acting upon the blood are often utilized by species which predate upon mammals, effectively disrupting hemostasis within the body leading to hemorrhagic shock or stroke [18,19]. Vertebrates possess a complex system of clotting factors which form a cascade, ultimately resulting in the formation of a fibrin clot, to prevent the occurrence of spontaneous bleeding or blood loss in the event of an injury [20,21]. Anticoagulant venoms act upon these clotting factors to prevent the formation of a fibrin clot. Anticoagulant venoms act by either inhibiting clotting enzymes (e.g., Factor Xa, or thrombin) or depleting the levels of the clotting factor fibrinogen either through destructive cleavage or by pseudo-procoagulant cleavage resulting in the formation of weak, unstable fibrin clots that rapidly degrade [20,21,22,23,24,25]. Alternatively, venoms can be procoagulant by activating a clotting factor in the cascade to ultimately result in endogenous thrombin being generated, with this thrombin in turn cleaving fibrinogen to form strong, stable fibrin clots [19,26,27,28,29]. 

Venoms which act on the coagulation cascade are particularly evident in species known to prey on mammals, as is the case for the giant *Bitis* species [30,31,32,33]. Comparatively the diets of the dwarf *Bitis* species are less known and therefore predictions about their venom effects cannot be made in an evidence-based manner. The desert species, *B. perengueyi* and *B. schneideri*, are known to feed primarily on lacertid lizards [4,5]. However, the dietary preferences of most of the dwarf *Bitis* species are understudied and it is assumed they are generalists that opportunistically feed on a variety of small mammals and reptiles [4,5]. Since the venom of most dwarf *Bitis* species have not been characterized, it is unknown how their venom relates to their diet and what evolutionary patterns their venom may play in the diversification within the *Bitis* genus. 

The two independent lineages of giant *Bitis* are the most widespread and medically significant of the *Bitis* species due to their distribution, abundance in farmland, and extremely large venom yields. The most well studied species (*B. arietans*, *B. gabonica* and *B. rhinoceros*) have been studied in-depth due to the potent anticoagulant and hemorrhagic effects of their venom, being responsible for a significant proportion of the mortality and morbidity of snakebites in Africa [4,5,30,33,34]. The venom of both lineages of giant *Bitis* species causes a variety of effects including spontaneous and persistent bleeding, incoagulable blood, hemorrhage, hypotension and rapid defibrinogenation [30,31,32,33]. Hemorrhage and oligemic shock caused by extravazation is the reported cause of death in many clinical accounts from envenomations by the giant *Bitis* species [4,5,33].

However, it is currently poorly understood if the severe hemorrhagic shock produced by giant *Bitis* species is only a result of internal bleeding due to tissue destruction and vascular damage, or if the venoms potently inhibit factors of the clotting cascade. Previous studies have identified some mechanisms of the anticoagulant action of the giant *Bitis* venoms. *B. arietans* venom has been identified to prevent clot formation of whole blood using methods such as thromboelastography (TEG) analysis [32]. Additionally, the venoms of *B. arietans* and *B. gabonica* have been shown to cause fibrinogen degradation and rapid defibrination in vivo, having direct proteolytic action upon fibrinogen yielding defective fibrin clots [32,33,35,36,37,38]. *B. nasicornis* and *B. parviocula* venom have also been shown to cause fibrinogen degradation, yet their venom has not been studied in detail [39,40]. Comparatively, the venom of dwarf *Bitis* species are known only for having cytotoxic effects and some neurotoxic effects [41,42,43,44]. Currently, no studies have investigated the functional effects of any dwarf *Bitis* venoms upon the clotting cascade. 

Overall the patterns of venom evolution within the *Bitis* genus are poorly understood and the effects of the crude venom of many species within the genus are uncharacterized. Thus, this study aimed to determine the evolution of coagulotoxic venom across the *Bitis* genus whilst understanding the clinical mechanisms of their venoms upon blood coagulation. There were two competing hypotheses tested, that the venom was either ancestrally procoagulant or ancestrally anticoagulant. These hypotheses were based upon the knowledge that the sister genus *Proatheris* possesses procoagulant venom [45,46]. However the two morphologically convergent giant *Bitis* lineages (*B. arietans* and the clade consisting of *Bitis gabonica*, *B. nasicornis*, *B. parviocula* and *B. rhinoceros*) are known to possess anticoagulant venom effects. Therefore this study will test whether anticoagulant or procoagulant venom is the plesiotypic (basal) condition and secondly if procoagulant venom is shown to be the basal condition, and whether the two independent lineages of giant species are linked to the apotypic (derived) state of anticoagulant venoms.

## 2. Results

### 2.1. Coagulation Assays 

**Figure 1:**
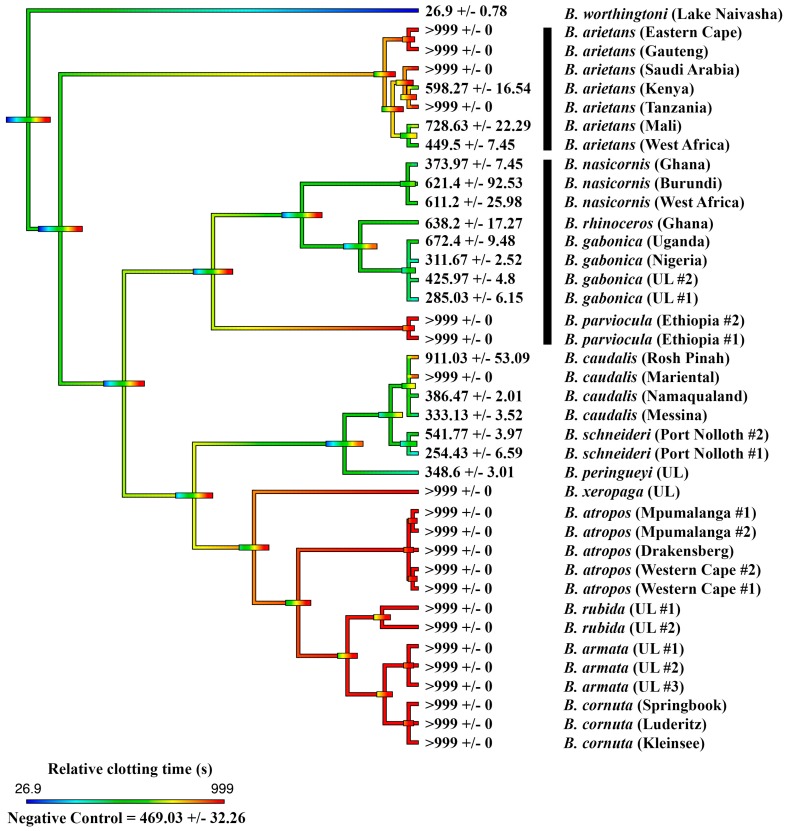
**Ancestral state reconstruction of coagulant venom effects on plasma clotting times within the *Bitis* genus.** Reconstruction of the ancestral state of the coagulotoxic effect of the venom of fourteen *Bitis* species upon the clotting ability of recalcified plasma, showing the time until clot formation and standard error of the mean. The spontaneous clotting of the recalcified plasma was 410.70 ± 9.96 sec. Warmer colors represent inhibition of clotting in plasma (anticoagulant venom effect) and cooler colors represent activation of clotting in plasma (procoagulant venom effect). Maximum machine reading time is 999 s, all means of >999 are venoms for which the clotting time ran to the maximum machine reading time. UL = unknown locality. Horizontal bars indicate 95% confidence intervals for the estimate at each node. Note: due to the dynamic nature of venoms, the bars rapidly become broad as one moves down the tree. Vertical black bars indicate the two independent lineages of giant species. Organismal phylogeny is based upon Alencar et al. (2016) and Barlow et al. (2019) [6,9].

**Figure 2:**
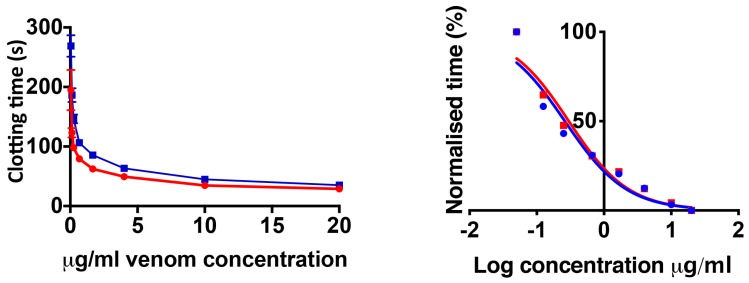
**Concentration–response curve showing the procoagulant effect of *B. worthingtoni* and relative efficacy of the South African Institute for Medical Research (SAIMR) antivenom.***B. worthingtoni* concentration dilution curve and logarithmic depiction, of both the venom-only assay condition (red line) and incubation with antivenom assay (blue line), showing the negligible effect of SAIMR polyvalent antivenom at neutralizing the effects of *B. worthingtoni* venom. Negative control values were 410.7 ± 9.96 s, which represents the time recalcified plasma spontaneously clots under natural conditions. Data points are *n* = 3 mean and standard error of the mean. Note: for most data points the error bars are shorter than the size of the symbol. UL = unknown locality.

The effects upon plasma were initially determined for all species, showing extreme diversity of venom effects within the genus. Only one species, *B. worthingtoni*, was determined to have procoagulant venom (Figure 1), whereby it rapidly clotted the plasma (28.93 ± 3.54 sec) at a rate much faster than that of the spontaneous clotting of recalcified plasma (410.70 ± 9.96 sec). The efficacy of the South African Institute for Medical Research (SAIMR polyvalent in neutralizing the procoagulant effect of *B. worthingtoni* was tested and shown to be ineffective (Figure 2). As procoagulation has been shown to be a characteristic of *Proatheris* venoms, the sister genus to *Bitis*, this venom action was thus inferred as the basal condition for *Bitis* venoms. 

**Figure 3:**
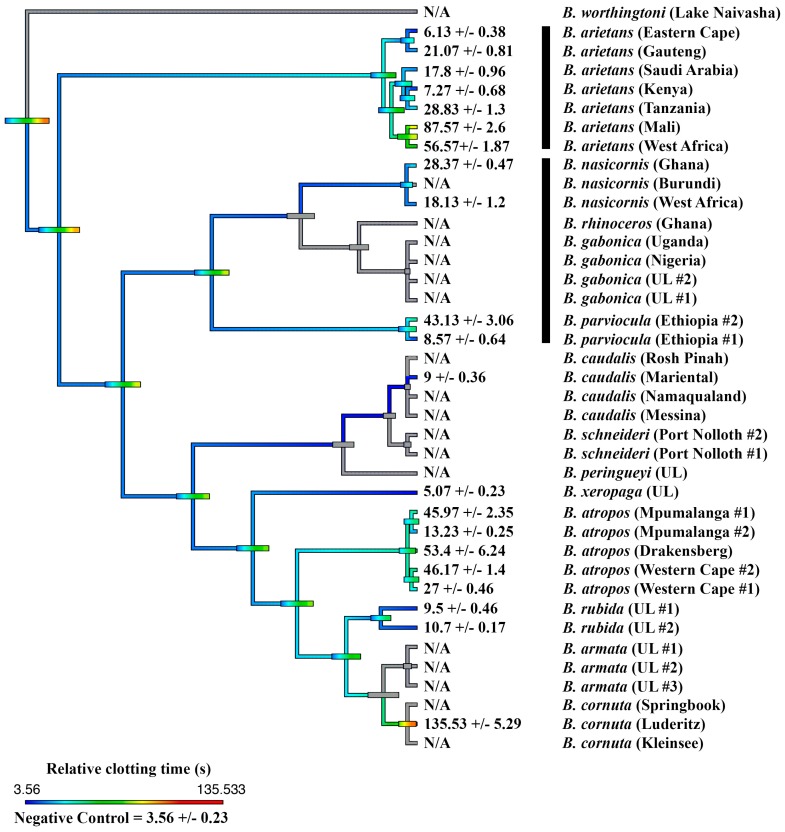
**Ancestral state reconstruction of fibrinogenolytic venom effects on clotting times within the *Bitis* genus.** Reconstruction of the ancestral state of the fibrinogen destroying effects of the venom of fourteen *Bitis* species upon the clotting ability of fibrinogen in the presence of thrombin after a 1-h incubation period, showing the time until clot formation and standard error of the mean. Warmer colors represent inhibition of clotting (fibrinogen destroying venom effect). N/A represents species for which the fibrinogen clotted during the 1-h incubation period, thus no measurement of time until clot formation in the presence of thrombin could be evaluated. UL = unknown locality. Horizontal bars indicate 95% confidence intervals for the estimate at each node. Note: due to the dynamic nature of venoms, the bars rapidly become broad as one moves down the tree. Vertical black bars indicate the two independent lineages of giant species. Organismal phylogeny is based upon Alencar et al. (2016) and Barlow et al. (2019) [6,9].

**Figure 4:**
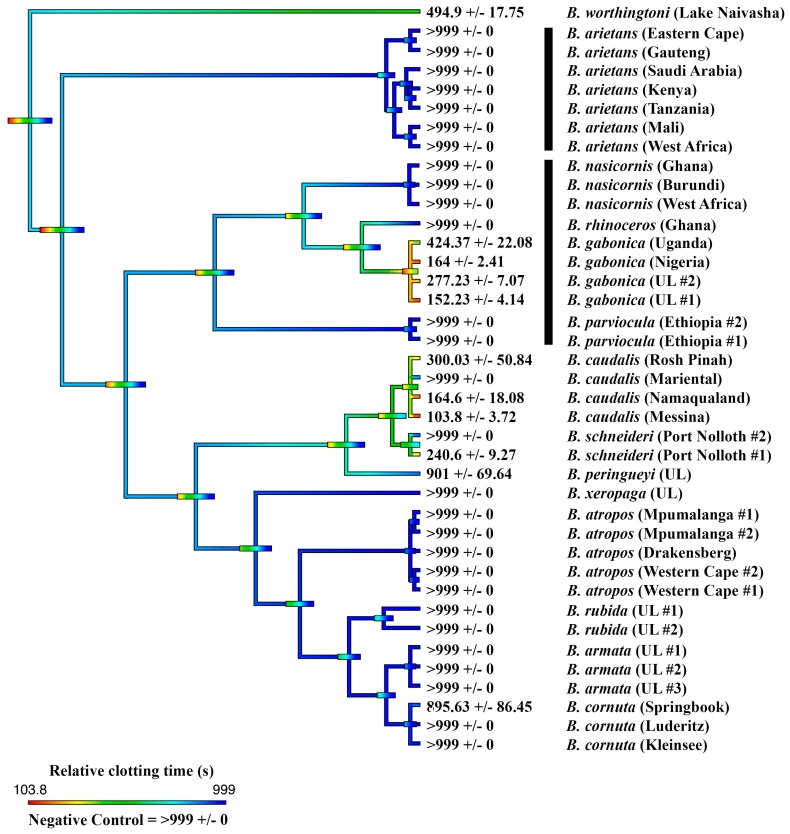
**Ancestral state reconstruction of fibrinogen clotting venom effects within the *Bitis* genus.** Reconstruction of the ancestral state of the coagulant effect of the venom of fourteen *Bitis* species upon the clotting ability of fibrinogen, showing the time until clot formation and standard error of the mean. Warmer colors represent clotting of fibrinogen (pseudo-procoagulant venom effect) and cooler colors represent no clotting of fibrinogen (no direct coagulant venom effect upon fibrinogen). Maximum machine reading time is 999 s, all means of >999 are venoms for which the clotting time ran to the maximum machine reading time. UL = unknown locality. Horizontal bars indicate 95% confidence intervals for the estimate at each node. Note: due to the dynamic nature of venoms, the bars rapidly become broad as one moves down the tree. Vertical black bars indicate the two independent lineages of giant species. Organismal phylogeny is based upon Alencar et al. (2016) and Barlow et al. (2019) [6,9].

**Figure 5:**
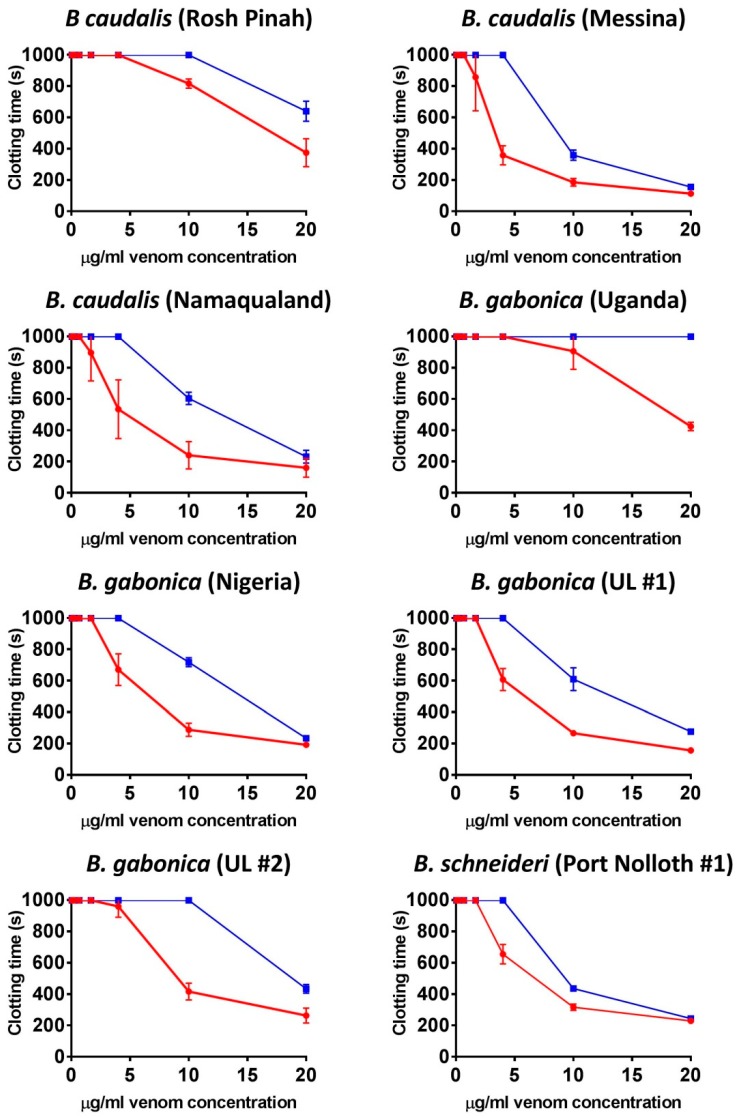
**Concentration–response curves for fibrinogen clotting activity and SAIMR antivenom efficacy.** Comparison of clotting curves showing the efficacy of SAIMR polyvalent antivenom on neutralizing the fibrinogen clotting activity of the three species which displayed a significant ability to clot fibrinogen. Red curves represent venom assay conditions and blue curves represent venom incubated with antivenom assay conditions. Negative control values were 999 ± 0 s. Data points are *n* = 3 mean and standard error of the mean. Note: for most data points the error bars are smaller than the line. UL = unknown locality.

**Figure 6:**
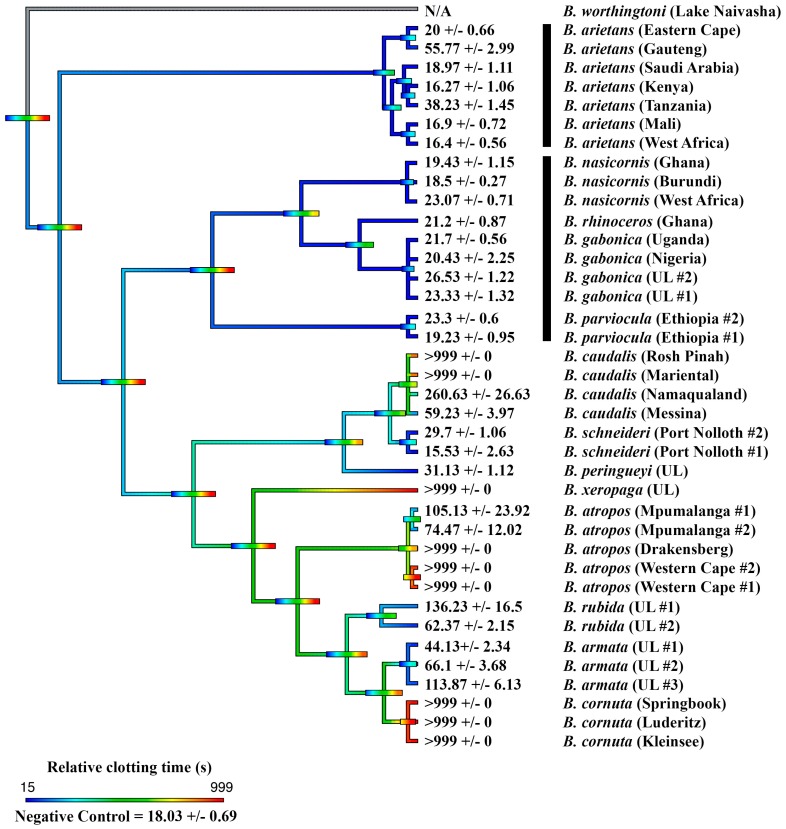
**Ancestral state reconstruction showing prothrombinase inhibition within the *Bitis* genus. Reconstruction** of the ancestral state of the prothrombinase inhibiting effect of the venom of fourteen *Bitis* species upon the clotting ability of recalcified plasma, showing the time until clot formation and standard error of the mean. Warmer colors represent inhibition of clotting in plasma after the addition of FXa to trigger clotting (prothrombinase inhibition) and cooler colors represent activation of clotting in plasma (absence of prothrombinase inhibition). Maximum machine reading time is 999 s; all means of >999 s are venoms for which the clotting time ran to the maximum machine reading time. N/A value for *B. worthingtoni* represents no value could be assessed for this species as it possesses procoagulant venom and could not be tested on this assay. UL = unknown locality. Horizontal bars indicate 95% confidence intervals for the estimate at each node. Note: due to the dynamic nature of venoms, the bars rapidly become broad as one moves down the tree. Vertical black bars indicate the two independent lineages of giant species. Organismal phylogeny is based upon Alencar et al. (2016) and Barlow et al. (2019) [6,9].

**Figure 7:**
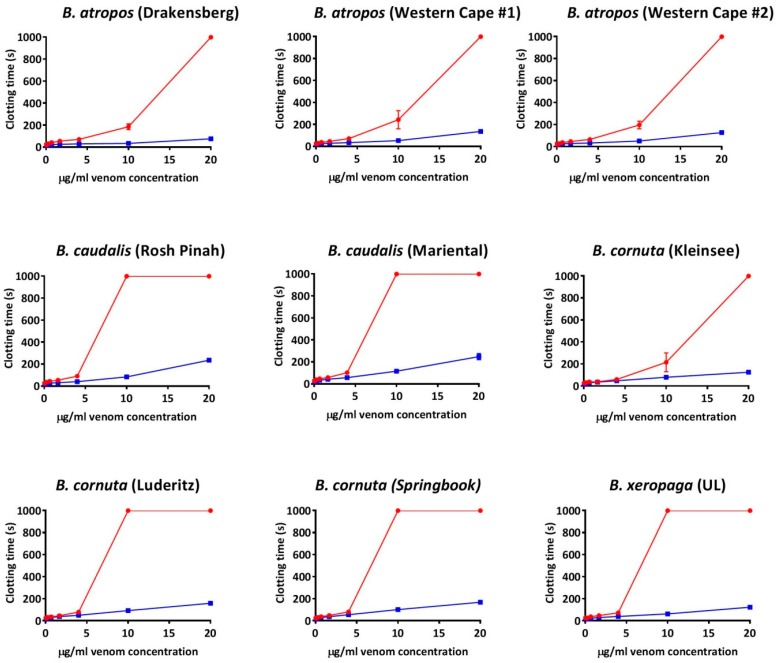
**Concentration–response curves for prothrombinase inhibition and SAIMR antivenom efficacy.** Comparison of clotting curves showing the efficacy of SAIMR polyvalent antivenom on neutralizing the prothrombinase inhibitory effects of the four species which displayed the most potent prothrombinase inhibition. Red curves represent venom assay conditions and blue curves represent venom incubated with antivenom assay conditions. Negative control values were 20.84 ± 0.51 s. Data points are *n* = 3 mean and standard error of the mean. Note: for most data points the error bars are smaller than the line. UL = unknown locality.

Thus, anticoagulant venom was shown to be a convergently amplified trait within four separate clades (Figure 1): *B. arietans* (with further amplification or secondary reductions within this species, each are equally plausible with the phylogenetic pattern present)*;* again in *B. parviocula*; again in the last common ancestor of the Mariental and Rosh Pinah populations of *B. caudalis;* and again in the last common ancestor of the clade consisting of the dwarf species *B. armata*, *B. atropos*, *B. caudalis*, *B. cornuta*, *B. rubida* and *B. xeropaga*. The other venoms did not differ appreciably from the plasma spontaneous clotting time of 410.7 ± 9.96 s (Figure 1). However, further testing using a different assay platform showed that different localities of *B. arietans* and *B. atropos*, *B. cornuta* (Luderitz locality) and *B. parviocula* had varying fibrinogenolytic effects upon fibrinogen, cleaving it in a destructive, non-clotting manner (Figure 3). In contrast, the ability to cleave and clot fibrinogen in a pseudo-procoagulant manner was found to be present in *B. caudalis, B. gabonica*, *B. schneideri* and *B. worthingtoni* (Figure 4) and SAIMR antivenom was shown to be effective at neutralizing the pseudo-procoagulant effect of these venoms (Figure 5). Inhibition of prothrombinase complex formation was demonstrated for venoms from *B. atropos* (Drakensberg and Western Cape localities), *B. caudalis* (Rosh Pinah and Mariental localities), and *B. cornuta* (all localities and *B. xeropaga* (Figure 6) with SAIMR polyvalent antivenom shown to be effective at neutralizing the prothrombinase complex formation inhibiting effects of all venoms, with the least efficacy for *B. atropos* (Mpumalanga #2) (Figure 7). 

### 2.2. Thromboelastography 

**Figure 8:**
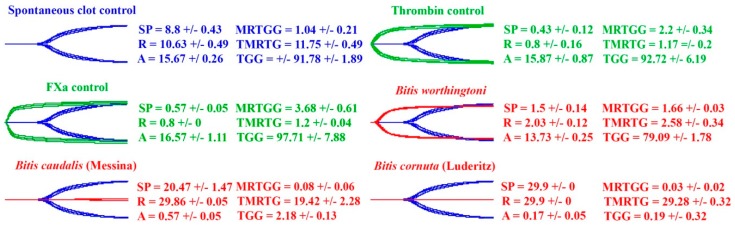
**Plasma thromboelastography.** Thromboelastography traces showing effects of venoms’ (20 µg/ml) ability to clot plasma relative to spontaneous clot control. Samples are three representative species of *Bitis* which possess procoagulant (*B. worthingtoni*), pseudo-procoagulant (*B. caudalis*) and anticoagulant (*B. cornuta*) venom. Blue traces represent spontaneous clot controls, green traces represent thrombin and FXa positive controls and red traces represent samples. SP = split point which is the time taken until clot begins to form (min). R = time to initial clot formation where formation is 2 mm+ (min). A (amplitude) = clot strength (mm). MRTGG = Maximum rate of thrombus generation (dynes/cm^2^/s). TMRTG = Time to maximum rate of thrombus generation (min). TGG = Clot strength (dynes/cm^2^). Overlaid traces are *n* = 3 for each set of controls and experimental conditions. Values are *n* = 3 means and standard deviations.

**Figure 9:**
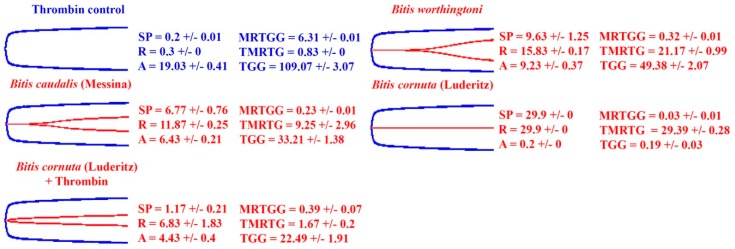
**Fibrinogen thromboelastography.** Thromboelastography traces showing effects of venoms’ (20 µg/ml) ability to clot fibrinogen relative to thrombin control. Samples are three representative species of *Bitis* which possess procoagulant (*B. worthingtoni*), pseudo-procoagulant (*B. caudalis*) and anticoagulant (*B. cornuta*) venom. Thrombin was added to *B. cornuta* (Luderitz) to regenerate a clot after the 30-min incubation of the venom with fibrinogen because no clot was detected. Blue traces represent spontaneous clot controls and red traces represent samples. SP = split point which is the time taken until clot begins to form (min). R = time to initial clot formation where formation is 2 mm+ (min). A (amplitude) = clot strength (mm). MRTGG = Maximum rate of thrombus generation (dynes/cm^2^/s). TMRTG = Time to maximum rate of thrombus generation (min). TGG = Clot strength (dynes/cm^2^). Overlaid traces are *n* = 3 for each set of controls and experimental conditions. Values are *n* = 3 means and standard deviations.

Thromboelastography results show there are three distinct effects upon fibrinogen from species within the *Bitis* genus: procoagulation through the generation of endogenous thrombin, resulting in strong, stable fibrin clots; anticoagulation through the pseudo-procoagulant cleavage of fibrinogen into weak, unstable fibrin clots; and anticoagulation through the destructive (non-clotting) cleavage of fibrinogen. The first mechanism would result in prey immobilization by stroke, while the latter two mechanisms result in the net depletion of fibrinogen levels, thereby promoting hemorrhagic shock. Thromboelastography testing upon plasma and fibrinogen showed that the venom of *B. worthingtoni* generates endogenous thrombin to form a strong plasma clot and also causes fibrinogen to clot directly (Figure 8 and Figure 9). Using thromboelastography, the venom of *B. caudalis* (Messina locality) was shown to prevent strong clotting in plasma compared to the spontaneous control (Figure 8). *B. caudalis* (Messina) venom was also shown to have a direct pseudo-procoagulant effect on fibrinogen, whereby the venom caused a weak fibrin clot to be formed (Figure 9). The venom of *B. cornuta* (Luderitz locality) was shown to prevent clotting in plasma but also had a destructive effect upon fibrinogen (Figure 8 and Figure 9). Fibrinogen in the presence of thrombin was also only able to form a very weak clot after being incubated for 30 min with *B. cornuta* (Luderitz) venom showing that the venom has a destructive effect upon a significant amount of the available fibrinogen (Figure 9). 

## 3. Discussion

This study assessed the coagulotoxic effects of venoms from fourteen species within the *Bitis* genus, and is the most detailed venom study conducted to-date on the genus. The strong procoagulant action of *B. worthingtoni* strongly suggests that procoagulant venom was the ancestral state of *Bitis* venom, shared between their last common ancestor with their sister genus *Proatheris*, which is morphologically very similar to *B. worthingtoni* in size, color, and pattern. This is the most parsimonious explanation, as the sister genus *Proatheris* possess procoagulant venom and *B. worthingtoni*, which is the plesiomorphic species within the *Bitis* genus, also possesses procoagulant venom (Figure 1). Uniquely, this is the first description of a *Bitis* species possessing procoagulant venom and also the first characterization of the functional venom effects for *B. worthingtoni*. SAIMR polyvalent antivenom was tested to determine its efficacy at neutralizing the procoagulant effect and was revealed to possess no significant neutralizing ability at preventing the potent procoagulant effect of *B. worthingtoni* venom. This indicates there is no cross reactivity between the *Bitis* species used in the antivenom immunizing mixture (*B. arietans* and *B. gabonica*) and the unique procoagulant function of *B. worthingtoni* venom (Figure 2). Therefore, patients envenomated by *B. worthingtoni* currently lack an effective treatment for these potentially lethal coagulotoxic effects. 

The majority of species in the *Bitis* genus were shown to possess anticoagulant venom and we conclude that anticoagulant venom is a trait that evolved subsequent to the split by *B. worthingtoni,* and was independently amplified on four separate occasions: *B. arietans* (with subsequent further amplification or secondary reductions within this species, each are equally plausible with the phylogenetic pattern present); again in *B. parviocula*; again in the last common ancestor of the Mariental and Rosh Pinah populations of *B. caudalis*; and again in the last common ancestor of the clade consisting of the dwarf species *B. armata*, *B. atropos*, *B. caudalis*, *B. cornuta*, *B. rubida* and *B. xeropaga* (Figure 1). Thus, anticoagulation is not a trait linked to gigantism in the *Bitis* lineage, as only some species or populations of giants have this trait, while the majority of dwarf *Bitis* also possess anticoagulant venom (Figure 1). Additionally, some species of giant *Bitis* (*B. gabonica*, *B. nasicornis* and *B. rhinoceros*) did not show significant anticoagulant activity upon preventing clot formation within recalcified plasma despite these species producing hemorrhage clinically. This clinical effect in these species may only be a result of damage to the vascular wall from destructive enzymatic toxins rather than direct anticoagulation. 

Within the *B. arietans* clade, subsequent to anticoagulant venom being a trait amplified at the base of the clade, there has been secondary amplification or reduction of this trait, with specific localities of this species possessing more potent anticoagulant venom than other populations. This is of clinical importance due to the significant medical importance of this species across Africa. Samples from Kenya and West Africa populations of *B. arietans* showed no significant inhibitory effect upon preventing spontaneous clotting of recalcified plasma (Figure 1). The *B. arietans* localities with the most potent anticoagulant effect were those from the Eastern Cape, Gauteng, Saudi Arabia and Tanzania localities (Figure 1). Geographical venom variation has been previously described in *B. arietans*, with proteomic variation in expression and activity of snake venom metalloproteases within the venom [40,41]. The venoms of the Gauteng, Mali, Saudi Arabia, Tanzania and West Africa localities for *B. arietans* also showed destructive ability upon fibrinogen, with the Mali locality identified as having the most potent effect upon the non-clotting, destructive cleavage of fibrinogen (Figure 3). This is consistent with previous studies that showed that *B. arietans* venom has fibrinogenolytic effects [24,28]. This indicates that the symptoms associated with *B. arietans* envenomations are caused by a combination of vascular wall damage and also direct destructive effects upon the blood coagulation factor fibrinogen. This direct effect upon fibrinogen was not present in the Eastern Cape or Kenya localities, further demonstrating the geographical variation in the functional effects for *B. arietans* venom. Thus, this work builds upon the body of knowledge, identifying that there is significant functional venom variation within *B. arietans* geographically. 

Within the *Bitis* clade comprised of the giant species *B. gabonica*, *B. nasicornis*, *B. parviocula* and *B. rhinoceros*, only *B. parviocula* venom significantly increased the clotting time of recalcified plasma (Figure 1). This shows that anticoagulant venom is not a trait that has coevolved with gigantism in this *Bitis* clade. Previous studies have identified that *B. parviocula* venom is potently hemorrhagic, as well as having strong effects upon platelets [33]. Assays with a 1-h incubation of *B. parviocula* venom with fibrinogen caused a delay in time until clot formation but did not prevent clotting and there was also intra-species variation between the two *B. parviocula* venoms tested (Figure 3). The effects of *B. parviocula* venom upon fibrinogen were like that of some of the sampled *B. arietans* localities (Figure 3). These results showing an effect of the venom upon fibrinogen are consistent with previous work assessing the fibrinogenolytic activities of this species’ venom [33]. Additional mechanisms of action responsible for the significant anticoagulant effects of *B. parviocula* venom were not conclusively determined in this study as it did not show any significant inhibitory effects on either the prothrombinase complex, thrombin, or Factor Xa inhibition assays conducted. 

The venoms of *B. gabonica*, *B. nasicornis* and *B. rhinoceros* did not have any anticoagulant effect upon the clotting time of recalcified plasma and thus it is likely that the majority of bleeding symptoms seen by envenomation patients are caused by the venom causing damage to the vascular wall rather than inhibiting coagulation. However, in a separate assay the venoms of all localities sampled of *B. gabonica* all clotted fibrinogen (Figure 4). This is identified as a pseudo-procoagulant effect, whereby the venom ineffectively cleaves fibrinogen to form a weak fibrin clots that are unstable and short-lived, thus reducing the amount of fibrinogen and therefore this may contribute to hemorrhagic shock in human bite victims. This result is consistent with previous studies which have identified that *B. gabonica* venom causes defibrination in envenomation patients [25]. This is further backed up by *B. gabonica* venom possessing thrombin-like, kallikrein enzymes of the type responsible for pseudo-procoagulant actions [42]. It was notable that venom of the closely related *B. rhinoceros* did not possess this trait. Three localities of the dwarf species *B. caudalis* and one locality of *B. schneideri* also possessed a potent pseudo-procoagulant effect whereby the venom causes fibrinogen to clot in a way which forms weak and unstable fibrin clots with short half-lives (Figure 4). SAIMR antivenom was also shown to be effective at neutralizing the pseudo-procoagulant effect of these venoms (Figure 5). Since the only *Bitis* species used in the immunizing mixture to make SAIMR antivenom are *B. arietans* and *B. gabonica*, this indicates the toxins responsible for this pseudo-procoagulant effect in *B. gabonica* are similar to *B. caudalis* and *B. schneideri*, allowing for the observed cross-reactivity of the antivenom (Figure 5). 

In addition to the pseudo-procoagulant effect observed in *B. caudalis* and *B. schneideri*, a wide range of novel coagulotoxic effects were identified in the dwarf *Bitis* venoms. Venoms from all populations of *B. armata*, *B. atropos*, *B. cornuta*, *B. rubida*, and *B. xeropaga* prevented plasma from clotting, as did two populations of *B. caudalis* (Figure 1). The venom of *B. caudalis* from the Namaqualand and Messina localities however possessed venom which had did not delay the clotting of recalcified plasma, which was comparable to the venoms of *B. perengueyi* and both populations of *B. schneideri* also having no strong effect upon the time until clot formation of recalcified plasma (Figure 1). However, in a separate assay these localities of *B. caudalis*, *B. perengueyi* and *B. schneideri* also targeted fibrinogen in a pseudo-procoagulant manner generating fibrin clots when incubated with fibrinogen (Figure 4). 

Unique to the anticoagulant dwarf *Bitis* species was the inhibitory action upon the formation of the prothrombinase complex. The most potent ability to prevent the formation of the prothrombinase complex was produced by the venoms of *B. atropos* (Drakensberg and Mpumalanga localities), *B. caudalis* (Rosh Pinah and Mariental localities), *B. cornuta* (Kleinsee locality), and *B. xeropaga* (Figure 6). SAIMR polyvalent antivenom was tested upon these venoms to determine its efficacy at neutralizing the inhibitory effects of these venoms and was shown to effectively neutralize the prothrombinase complex formation inhibitory action of all venoms (Figure 7). 

Notably, we identify functional geographical venom variation between *B. atropos* localities with the prothrombinase inhibiting activity present in the Drakensberg and Western Cape localities but absent from the Mpumalanga localities (Figure 6). *B. atropos* venom from all localities also displayed varying fibrinogenolytic potency when incubated with fibrinogen (Figure 3). Previous clinical studies of *B. atropos* envenomations have also suggested that this species may show geographical venom variation [35]. The anticoagulant effect seen on plasma by the Western Cape localities of *B. atropos* however is not likely solely due to their effect upon fibrinogen. The site of action which is targeted by the venoms of the species *B. armata* and *B. rubida* was also not able to be identified in this study. Further testing upon other factors of the clotting cascade which make up the extrinsic and intrinsic pathways is required to elucidate the mechanisms behind these venoms. 

Thromboelastography testing was also conducted upon three representative venoms in this study to confirm the three main coagulotoxic effects upon fibrinogen seen in the coagulation assays. *B. worthingtoni*, *B. caudalis* (Messina locality) and *B. cornuta* (Luderitz locality) represented three species in the study which had procoagulant (generation of endogenous thrombin with the subsequent formation of strong, stable fibrin clots), pseudo-procoagulant (cleavage of fibrinogen to form weak, unstable fibrin clots leading to depletion of fibrinogen levels), and anticoagulant (cleavage of fibrinogen in a destructive, non-clotting manner leading to depletion of fibrinogen levels and thus consequent reduced clotting ability). Thromboelastography testing upon plasma showed that the strength of the clot for *B. worthingtoni* was indicative of the generation of endogenous thrombin and a true procoagulant venom effect (Figure 8). However, *B. worthingtoni* venom also possessed the pseudo-procoagulant ability to weakly clot fibrinogen, showing this venom also possess toxins which have a pseudo-procoagulant effect (Figure 9). This indicates *B. worthingtoni* has multiple toxins present, acting together upon the clotting ability of plasma to disrupt hemostasis. However, as seen with other genera such as *Atractaspis* and *Bothrops*, the procoagulant speed of action was dramatically faster than the pseudo-procoagulant action and therefore this would dominate the effects upon the blood [21,23]. Using thromboelastography, *B. caudalis* (Messina) was also demonstrated to possess this pseudo-procoagulant effect, where fibrinogen is ineffectively cleaved to form a weak fibrin clot (Figure 9, but this venom did not have a true procoagulant effect and therefore depletion of fibrinogen would be the dominant effect upon the blood. In comparison *B. cornuta* (Luderitz) prevented clotting of fibrinogen and when thrombin was added after 30min only a weak clot (A = 4.43 +/− 0.4) was able to be formed compared to the control (A = 19.03 +/− 0.41) (Figure 9). This shows that the venom of *B. cornuta* (Luderitz) possesses a true destructive effect upon fibrinogen, reducing the amount of available fibrinogen which thrombin could act upon. Thus, the implementation of thromboelastography in addition to the coagulation assays conclusively showed three distinct coagulotoxic effects within the *Bitis* genus; procoagulation, pseudo-procoagulation and anticoagulation. 

## 4. Conclusions

In conclusion, this study elucidated both the phylogenetic patterns of venom evolution within the *Bitis* lineage as well as describes novel mechanistic actions within the genus for several understudied species. This study revealed that procoagulant venom is the plesiotypic (basal) condition within the *Bitis* genus, but that this trait is retained only in *B. worthingtoni.* Future work should be undertaken to determine mechanism of action of the *B. worthingtoni* procoagulation (eg activation of Factor X or prothrombin). This study also showed that a diversity of anticoagulant venom actions have evolved within other species, including differential amplification/secondary reduction of some functions. Within the genus we also identified a wide range of anticoagulant mechanisms present including cleaving fibrinogen in a pseudo-procoagulant manner to form weak unstable fibrin clots that are rapidly degraded, destructive cleavage of fibrinogen leading to a depletion of fibrinogen levels, and also inhibition of formation of the prothrombinase complex. The anticoagulant mechanisms for *B. armata*, *B. rubida* and the Western Cape locality of *B. atropos* were not fully identified, however, and future work should endeavor to resolve the mechanisms behind the anticoagulant effect of these venoms including inhibition of clotting enzymes such as FIXa, FXa, FXIa, and thrombin. In addition to the contribution to the body of knowledge regarding venom evolution, this study also has clinical implications. Only the anticoagulant actions (inhibition of the prothrombinase-complex formation, and depletion of fibrinogen levels either through pseudo-procoagulant cleavage or non-clotting, destructive fibrinogen cleavage) are well neutralized by the sole available antivenom. In contrast, the procoagulant action of *B. worthingtoni* was not neutralized by the antivenom, indicating limited treatment options for this pathophysiological effect. These results therefore have real-world applications in the design of clinical management strategies, as the results demonstrate that these venoms will produce differential coagulopathy in the envenomed patient.

## 5. Materials and Methods 

### 5.1. Venoms and Reagents

This study aimed to characterize the venom from fourteen of the seventeen species within the medically significant *Bitis* genus. For samples from species where the geographical locality of founding stock was unknown the abbreviation UL was used. *B. arietans* (Saudi Arabia, Kenya, Mali, Tanzania and West Africa localities) and *B. nasicornis* (Burundi and West Africa localities) venoms were supplied by Latoxan (Portes-les-Valence, France). All other venom samples were captive specimens sourced from a long-term cryogenic research collection. Venoms were lyophilized, frozen and stored at −80 °C. Venom working stock solutions (50% glycerol and 50% deionized water) were made at a concentration of 1 mg/mL and stored at −20 °C until required to preserve enzymatic activity. Bovine Factor Xa (Stago catalog #00311), calcium (Stago catalog # 00367), phospholipid (Stago catalog #00597) and Owren–Koller buffer (Stago Catalog # 00360) and thrombin (Stago catalog # 00611) were supplied by Stago. Human plasma was supplied by the Red Cross Blood Service (Research Supply Agreement 18-03QLD-09), pooled, and stored at −80 °C. All work was undertaken under Biosafety Approval #IBC134BSBS2015 (1 January 2015) and Human Ethics Approval # 2016000256 (1 June 2016).

### 5.2. Coagulation Assays

Coagulation assays were carried out on a Stago STA-R Max coagulation analyzing robot (Stago, France). Pooled frozen human plasma was thawed and warmed to 37 °C for 5 min in a water bath before being placed in the Stago STA-R Max machine. To determine the coagulotoxic effects of each sample, venom from a 1 mg/mL glycerol stock solution was manually diluted in Owren–Koller buffer (isotonic saline) to make a 0.1 µg/µL working solution. A total of 50 µL of 0.1 µg/µL venom was added by the STA-R Max to 50 µL calcium, 50 µL phospholipid and 25 µL Owren–Koller Buffer, then incubated for 120 s before the robot shook the sample to mix the reagents and then added 75 µL human plasma and clot formation immediately measured. Time until clot formation was measured within the cuvette directly after the addition of the human plasma. Calcium and phospholipid were added to the coagulation assays to replicate the in vivo conditions present in the human body. Experiments were carried out in triplicate at each concentration point. Assays in which a clot had not formed after 999 s were automatically stopped by the robot, as this is the machine maximum measurement time. Negative controls were conducted with stocks of 50% Owren–Koller buffer and 50% glycerol used in replacement of venom, to represent the time healthy plasma spontaneously clots. For any species that activated the clotting cascade, decreasing time until clot formation, antivenom conditions where run with 5% concentration of South African Institute for Medical Research (SAIMR) polyvalent antivenom (Batch Number BF00846, South Africa Institute for Medical Research, South Africa) used in place of the 25 µL Owren–Koller buffer (as this was 1/10 the concentration, the final antivenom concentration in the reaction was 0.5%). To identify the target in the clotting cascade which anticoagulant venoms were acting upon, either plasma or individual factors of the clotting cascade were incubated with the sample venom. SAIMR antivenom was tested against venoms on specific assays which showed significant results. The incubation step is included as it allows for the venom to bind and inhibit its target. Table 1 details the methodology used for each of the inhibitory assays conducted. 

### 5.3. Thromboelastography (TEG) 

To determine the effect of *Bitis* venoms upon the viscoelastic clot properties of the fibrin clots TEG was utilized to assess the venoms of three functionally representative species of *Bitis* (one procoagulant, one pseudo-procoagulant and one anticoagulant species, chosen based upon the STA-R Max result patterns). *B. worthingtoni* was selected as the procoagulant species, *B. caudalis* (Messina locality) as the pseudo-procoagulant species and *B. cornuta* (Luderitz locality) as the anticoagulant species. The venom of each species was tested individually upon plasma and fibrinogen. For assays testing the effect of the venom upon fibrinogen or plasma the following conditions assay conditions were run; 7 µL of venom (1 mg/mL in 50% glycerol) was added to 72 µL calcium, 72 µL phospholipid, 20 µL Owren–Koller buffer and 189 µL human fibrinogen (4 mg/mL) from Sigma Aldrich or human plasma. For assays upon fibrinogen, if no clot was formed after 30 min, 7 µL of thrombin was added to determine if any fibrinogen was remaining to allow a clot to form. Fibrinogen assay controls were conducted in triplicate using 7 µL of thrombin in replacement of venom. Plasma assays negative controls were conducted in triplicate using 7 µL of Owren–Koller buffer: glycerol (50:50) in replacement of venom to represent spontaneous clotting. Positive controls using either 7 µL of Factor Xa or thrombin in replacement of venom were also conducted for the plasma assay.

### 5.4. Statistics 

Venom and antivenom concentration–response curves and area under the curve (AUC) values were analysed using Prism 7.0 software (GraphPad Software Inc., La Jolla, CA, USA, 2019). *t*-Tests were used to test if venom and antivenom AUC values being compared were significantly different. For all statistical tests *p*-values ≤ 0.05 were considered statistically significant. Phylogenetic analysis was conducted to determine ancestral states within the *Bitis* lineage [26,28]. The phylogenetic tree used to assess evolutionary patterns across the genus is based upon Alencar et al. (2016) and Barlow et al. (2019) and was imported into R for evolutionary analysis using the APE package [6,9,47]. This analysis allowed for the estimation of the ancestral states for the venom of the *Bitis* genus using maximum likelihood, as implemented in the contMap function of the R package phytools [48] (Supplementary File 1). 

## Figures and Tables

**Table 1:** Clotting cascade inhibition assay conditions.

Factor Xa inhibition assay	Step 1: 50 µL 0.1 µg/mL venom (1 mg/mL 50% glycerol stock diluted with Owren–Koller (OK) Buffer (Stago catalog # 00360) + 50 µL 0.025 M calcium (Stago catalog # 00367) + 50 µL phospholipid (Stago catalog #00597) + 25 µL Factor Xa (Stago catalog #00311). Step 2: 120 s incubation. Step 3: Addition of 75 µL plasma.
Thrombin inhibition assay	Step 1: 50 µL 0.1 µg/mL venom (1 mg/mL 50% glycerol stock diluted with OK Buffer + 50 µL 0.025 M calcium + 50 µL phospholipid + 25 µL thrombin. Step 2: 120 s incubation. Step 3: Addition of 75 µL 4 mg/mL fibrinogen.
Fibrinogen destruction assay	Step 1: 50 µL 0.1 µg/mL venom (1 mg/mL 50% glycerol stock diluted with OK Buffer + 50 µL 0.025 M calcium + 50 µL phospholipid + 75 µL of 4 mg/mL fibrinogen. Step 2: 1-h incubation. Step 3: Addition of 25 µL thrombin (Stago Liquid Fib kit catalog # 00673).
Fibrinogen clotting assay	Step 1: 50 µL 0.1 µg/mL venom (1 mg/mL 50% glycerol stock diluted with OK Buffer + 50 µL 0.025 M calcium + 50 µL phospholipid + 25 µL OK Buffer. Step 2: 120 s incubation. Step 3: Addition of 75 µL of 4 mg/mL fibrinogen.
Fibrinogen clotting assay (antivenom assay conditions)	Step 1: 50 µL 0.1 µg/mL venom (1 mg/mL 50% glycerol stock diluted with OK Buffer + 50 µL 0.025 M calcium + 50 µL phospholipid + 25 µL of 5% concentration of SAIMR polyvalent antivenom diluted with OK Buffer. Step 2: 120 s incubation. Step 3: Addition of 75 µL 4 mg/mL fibrinogen.
Prothrombinase complex inhibition assay	Step 1: 50 µL 0.1 µg/mL venom (1 mg/mL 50% glycerol stock diluted with OK Buffer + 50 µL 0.025 M calcium + 50 µL phospholipid + 75 µL plasma. Step 2: 120 s incubation. Step 3: Addition of 25 µL Factor Xa (Stago catalog # 00311).
Prothrombinase complex inhibition assay (antivenom assay conditions)	Step 1: 25 µL 0.2 µg/mL venom (1 mg/mL 50% glycerol stock diluted with OK Buffer + 75 µL of [50 µL 0.025 M calcium +25 µL of 5% concentration of SAIMR polyvalent antivenom diluted in OK Buffer] + 50 µL phospholipid + 75 µL plasma. Step 2: 120 s incubation. Step 3: Addition of 25 µL Factor Xa.

## Supplementary Materials

The following are available online at https://www.mdpi.com/2072-6651/11/7/422/s1, Supplementary File 1: phytools script.
