# Supplementary material for: Venomous Landmines: Clinical Implications of Extreme Coagulotoxic Diversification and Differential Neutralization by Antivenom of Venoms within the Viperid Snake Genus Bitis
Source: Toxins (Basel). 2019 Jul 19;11(7):422. doi: 10.3390/toxins11070422 (PMC6669450; doi:10.3390/toxins11070422)
Supplement: Supplementary file 1 [file toxins-11-00422-s001.pdf]

Supplementary Materials: Venomous Landmines: Clinical Implications of Extreme Coagulotoxic Diversification and Differential Neutralization by Antivenom of Venoms within the Viperid Snake Genus Bitis

Nicholas J. Youngman, Jordan Debono, James S. Dobson, Christina N. Zdenek, Richard J. Harris, , Bianca op den Brouw, Francisco C. P. Coimbra, Arno Naude, Kristian Coster, Eric Sundman, Ralph Braun, Iwan Hendrikx and Bryan G. Fry

Phytools script

```
>library(ape)
>library(maps)
>library(phytools)
># senci.contMap is a slight modification of errorbar.contMap that trims 95% CIs of
ancestral state reconstructions to a sensible range, e.g., for traits bound between 0 and 1
    # Example of code for implementing it would be as follows (lines separated by
    semicolons): pasr<-contMap(tree,mapdat,plot=F,lims=c(0,1)) ;
    plot(setMap(pasr,invert=T)); senci.contMap(setMap(pasr,invert=T),mini=0,maxi=1)
    senci.contMap<-function(obj,...){
    if (hasArg(x))
    x <- list(...)$x
    else x <- setNames(sapply(1:Ntip(obj$tree), function(x, obj) {
    ii <- which(obj$tree$edge[, 2] == x)
    ss <- names(obj$tree$maps[[ii]][length(obj$tree$maps[[ii]])])
    obj$lims[1 ] + as.numeric(ss)/(length(obj$cols) − 1) *
    diff(obj$lims)
    }, obj = obj), obj$tree$tip.label)
    if (hasArg(scale.by.ci))
    scale.by.ci <- list(...)$scale.by.ci
    else scale.by.ci <- TRUE
    if (hasArg(lwd))
    lwd <- list(...)$lwd
    else lwd <- 14
    tree <- obj$tree
    aa <- fastAnc(tree, x, CI = TRUE)
    if (hasArg(min))#added lines here
    for (i in 1:length(aa$CI95[,1])){ #added lines here
    aa$CI95[i,1]<-ifelse(aa$CI95[i,1]<list(...)$min,list(...)$min,aa$CI95[i,1]) #added lines
    here
    } #added lines here
```

```
        else aa$CI95[,1]<-aa$CI95[,1] #added lines here
        if (hasArg(max)) #added lines here
        for (i in 1:length(aa$CI95[,2])){ #added lines here
        aa$CI95[i,2]<-ifelse(aa$CI95[i,2]>list(...)$max,list(...)$max,aa$CI95[i,2]) # added
        lines here
        } #added lines here
        else aa$CI95[,2]<-aa$CI95[,2] #added lines here
        xlim <- range(aa$CI95)
        if (xlim[2] > obj$lims[2] || xlim[1] < obj$lims[1]) {
        cat(paste(" -----\n The range of the contMap object, presently (",
        round(obj$lims[1], 4), ",", round(obj$lims[2], 4),
        "), should be equal to\n or greater than the range of the CIs on ancestral states: (",
        round(xlim[1], 4), ",", round(xlim[2], 4), ").\n",
        sep = ""))
        cat(paste(" To ensure that your error bars are correctly plotted, please recompute
        your\n",
        " contMap object and increase lims.\n -----\n",
        sep = ""))
        }
        d <- diff(obj$lims)
        if (scale.by.ci) {
        v <- aa$CI95[, 2] - aa$CI95[, 1]
        v <- v/max(v)
        }
        else v <- rep(0.5, tree$Nnode)
        n <- length(obj$cols) - 1
        lastPP <- get("last_plot.phylo", envir = .PlotPhyloEnv)
        h <- max(nodeHeights(tree))
        for (i in 1:tree$Nnode) {
        ii <- round((aa$CI95[i, 1] - obj$lims[1])/d * n)
        jj <- round((aa$CI95[i, 2] - obj$lims[1])/d * (n + 1))
        cols <- obj$cols[ii:jj]
        add.color.bar(leg = 0.1 * h * v[i], cols = cols, prompt = FALSE,
        x = lastPP$xx[i + Ntip(tree)] - 0.05 * h * v[i],
        y = lastPP$yy[i + Ntip(tree)], title = "", subtitle = "",
        lims = NULL, lwd = lwd)
        }
        }
>data<-read.csv(file.choose())
>dat<-data
>mapvar<-dat$var
```

```
>names(mapvar)<-dat$species
>tree<-read.tree(file.choose())
>tree<-chronos(tree)
>asr<-contMap(tree,mapvar,plot=F)
>plot(setMap(asr,col=c(2,7,3,5,4)),lwd=3)
>senci.contMap(setMap(asr,col=c(2,7,3,5,4)),lwd=6,min=[minimum value from the
dataset],max=[minimum value from the dataset])
```
